# Immunoregulatory effects of RGMb in gut inflammation

**DOI:** 10.3389/fimmu.2022.960329

**Published:** 2022-11-07

**Authors:** Magdiel Pérez-Cruz, Bettina P. Iliopoulou, Katie Hsu, Hsin-Hsu Wu, Tom Erkers, Kavya Swaminathan, Sai-Wen Tang, Cameron S. Bader, Neeraja Kambham, Bryan Xie, Rosemarie H. Dekruyff, Gordon J. Freeman, Everett Meyer

**Affiliations:** ^1^ Division of Blood and Marrow Transplantation, Department of Medicine, Stanford University School of Medicine, Stanford, CA, United States; ^2^ Department of Developmental biology, Stanford University School of Medicine, Stanford, CA, United States; ^3^ Department of Pediatrics, Stanford University School of Medicine, Stanford, CA, United States; ^4^ Department of Medical Oncology, Dana-Farber Cancer Institute, Harvard Medical School, Boston, MA, United States

**Keywords:** RGMB, GvHD, gut inflammation, colitis, immunoregulation

## Abstract

Graft-versus-host disease (GvHD) is a major complication after allogeneic hematopoietic cell transplantation (HCT). Current strategies to prevent GvHD with immunosuppressive drugs carry significant morbidity and may affect the graft-versus-tumor (GVT) effect. Inflammatory bowel disease (IBD) is an intestinal inflammatory condition that affects more than 2 million people in the United States. Current strategies to prevent colitis with immunosuppressive drugs carry significant morbidity. Recently, Repulsive Guidance Molecule b (RGMb) has been identified as part of a signaling hub with neogenin and BMP receptors in mice and humans. In addition, RGMb binds BMP-2/4 in mice and humans as well as PD-L2 in mice. RGMb is expressed in the gut epithelium and by antigen presenting cells, and we found significantly increased expression in mouse small intestine after total body irradiation HCT conditioning. We hypothesized that RGMb may play a role in GvHD and IBD pathogenesis by contributing to mucosal inflammation. Using major-mismatched HCT mouse models, treatment with an anti-RGMb monoclonal antibody (mAb) that blocks the interaction with BMP-2/4 and neogenin prevented GvHD and improved survival compared to isotype control (75% versus 30% survival at 60 days after transplantation). The GVT effect was retained in tumor models. Using an inflammatory bowel disease dextran sulfate sodium model, treatment with anti-RGMb blocking monoclonal antibody but not isotype control prevented colitis and improved survival compared to control (73% versus 33% at 21 days after treatment) restoring gut homeostasis. Anti-RGMb mAb (9D1) treatment decreased IFN-γ and significantly increased IL-5 and IL-10 in the gut of the treated mice compared to the isotype control treated mice.

## Introduction

Therapeutics that interrupt major signaling pathways involved in T cell activation, proliferation and polarization are the backbones of modern strategies to prevent transplantation rejection (1, 2). A number of studies implicate the bone morphogenetic proteins (BMPs) and neogenin pathways as drivers of innate and adaptive inflammation, but it is unclear if these molecules are involved in transplantation tolerance. RGMb serves as a co-receptor for BMP2 and BMP4, in complex with neogenin and in mouse, can also bind the immune coinhibitory molecule PD-L2 (CD273) (3–6), and these interactions can regulate pulmonary mucosal immunity (6, 7). We hypothesized that the RGMb signaling hub may also play an important role in gut mucosal immunity, particularly in the context of HCT, and the pathway could represent a potential new therapeutic target.

BMPs have diverse roles in many physiologic and pathologic processes, including cell proliferation, differentiation, and apoptosis (8–10). BMP signaling has an established role in embryonic homeostasis but is also required for postnatal murine hematopoietic stem cell self-renewal (11). RGMb is a member of the RGM family, which consists of RGMa, RGMb (Dragon), and RGMc (hemojuvelin). RGMb has a well-elucidated role in neural development, with RGMb knock-out mice dying 2-3 weeks after birth (12). While RGMb neural expression in adult mice is limited to the basal ganglia and pituitary, the protein is expressed more broadly in the gut, bone, heart, lung, liver, kidney, testis, ovary, uterus, epididymis, and pancreas of adult mice (13, 14), and humans (15, 16). Recent studies have shown that RGMb is also expressed by immune cell subsets including macrophages (6). RGMb can regulates IL-6 expression *via* the BMP pathway in macrophages mediated by the p38 MAPK and Erk1/2 pathways but not by the Smad1/5/8 pathway (12).

All three RGM members function as coreceptors that enhance BMP signaling, which is generally proliferative (4). The crystal structure and binding regions of RGMb have been reported and revealed a complex signaling hub (17). RGMb can bind to neogenin and either BMP2 or BMP4 in a single complex and the interaction of RGMb with BMPs, PD-L2, and to a lesser extent, neogenin can be blocked by RGMb mAb 9D1 (6). PDL-2 mAb 2C9 blocks PD-L2-RGMb but not PD-L2-PD-1 interactions (6). While PD-L2 binding to RGMb occurs in mice, this has not been observed in humans, but soluble CTLA-4 (CD152) has been proposed to interact with RGMb (18, 7). A substantial proportion of RGMb is located inside the cell (7) where RGMb/Neogenin can form complexes with BMP type II and type I receptors, thereby increasing BMP signaling (19, 20).

Since BMP2, BMP4, and neogenin can be expressed by T cells and have been shown to regulate the activation of naïve T cells (21), it is possible that RGMb may participate to regulate T cell proliferative responses. Alternatively, RGMb may regulate T cells through its interaction with PD-L2 expressed on antigen-presenting cells or other immune cells. This signaling hub could therefore play an important role in transplantation tolerance and autoimmunity.

Donor T cells play an important role in HCT, with a large body of evidence supporting the model that T cells, and naive T cells, can drive graft-versus-host disease (GvHD), a serious complication in which donor T cells recognize and attack host epithelial skin, liver, and gut tissue. HCT conditioning alters the recipient immune environment in ways that can contribute to exacerbate GvHD. For example, conditioning can cause the upregulation of inflammatory mediators such as TNF-α and IFN-γ in the gut mucosal surface, which is a major site of GvHD. Importantly, RGMb expression levels are high in the gut (22) and increase with gut damage, suggesting a potential role for this molecule in gut immunity and as a possible driver of gut GvHD. RGMb deficiency significantly altered the diversity of gut microbiota and also induced dysbiosis (23). In this study, we investigated a role for RGMb in donor T cell function and GvHD amelioration.

Inflammatory bowel disease (IBD) is a chronic and multifactorial gastrointestinal inflammatory condition that is clinically categorized as ulcerative colitis (UC) or Crohn’s disease (CD). The initiation and progression of human IBDs are reliant on the dysregulation of complex interactions among genetic, environmental, and immune factors, as well as physical barriers within the intestinal mucosa. The physical barrier between the external environment and internal tissue is the first line of defense against microbial pathogens, toxins, and other environmental factors (24). This protective barrier is provided by the inner lining of the intestine, a single-cell layer of intestinal epithelial cells (IECs), and their specialized subtypes (e.g., Paneth, goblet, or enteroendocrine cells) (25). IECs serve an essential role as regulators of mucosal immune responses (26). By contrast, Rauch et al. used mice with conditional deletion of Ifnar1 in DCs or in myeloid cells. The study showed a protective effect of IFN-I by suppressing IL-1 production during inflammation of the gut. Altogether, IFN-I activates and orchestrates different programs to keep inflammation under control. Importantly, RGMb expression levels are high in the gut (22) and increase with gut damage, suggesting a potential role for this molecule in gut immunity and as a possible driver of gut IBD.

In order to understand how broad a role RGMb might have in mucosal immunity, in addition to GvHD, we evaluated IBD. The present study investigated the effect of anti-RGMb blocking antibody on experimental colitis induced by dextran sulphate sodium (DSS) in C57BL/6 mice, with the aim to characterize the colonic inflammatory response at the cellular and molecular level.

In both GvHD and IBD models, we found that 9D1 RGMb mAb blockade of the neogenin/BMP-2/4 binding site ameliorates gut tissue inflammation and disease.

## Materials and methods

### Mice

Eight-week-old BALB/CJ, NSG (NOD.Cg-Prkdc^scid^ Il2rg^tm1Wjl^/SzJ), C5BL/6 mice were purchased from Jackson Laboratories (Sacramento, CA). Luciferase-expressing (luc) C57BL/6 mice were created as described previously (27). Mice were maintained under a 12-hours light-dark cycle and were fed with a standard laboratory diet. The inhalational anesthetic isoflurane was administered during bioluminescence imaging (BLI). All studies were approved by Institutional Animal Care and Use Committee of Stanford University.

### Bone marrow and cell transplantation

BALB/c mice were conditioned with total body irradiation (TBI) (2x400 cGy, 200 kV X-ray source; Kimtron), injected with 5x10^6^ T-cell depleted donor bone marrow (TCD-BM) cells combined with 1x10^6^ CD4^+^/CD8^+^ T cells from C57BL/6 or luc^+^ C57BL/6 mice. For analysis of the GVT effect, 1x10^4^ of Luciferase-expressing mouse B cell A20 lymphoma cells were injected i.v. with TCD-BM on day 0 into C57BL/6 mice after TBI. In a second experiment, 500 BCL_1_ leukemia cells were injected i.v. with TCD-BM on day 0 into C57BL/6 after TBI (250 cGy).

NSG (NOD-SCID IL2 receptor gamma null) mice received 250 cGy total body irradiation before injection. Human PMBC (5x10^6^ cells/mouse) were injected *via* the tail vein to create graft-versus-host disease. Anti-RGMB (307.9D1, rat IgG2a, k and 506.2E11, rat IgG2a, k) (6) or isotype control antibody (2A3, rat IgG2a, k; BioXcell) was given at indicated time points.

### Induction of acute DSS-induced colitis

**Figure 1:**
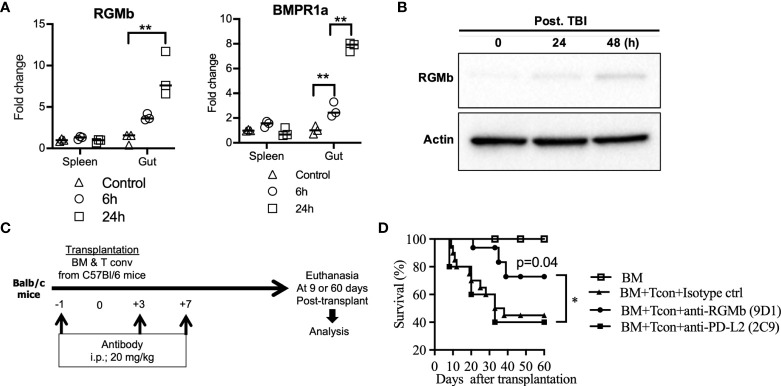
Treatment with anti-RGMb antibody 9D1 reduces GvHD. **(A)** To analyze the effect of TBI on RGMb expression in spleen and gut, RGMb and BMPR1a mRNA expression were quantified by qPCR at 6 and 24h after TBI and **(B)** RGMb protein expression in the gut was examined by western blotting at 24 and 48h after TBI. One-way ANOVA comparisons **(C)** To assess the capacity of anti-RGMb-(BMP2,4, NEO1 & PD-L2 blocking) or anti-PD-L2 (RGMb but not PD-1 blocking) antibody treatment to prevent GvHD, mice were injected with 9D1 or 2C9 antibody (20 mg/Kg; i.p.) at the indicated times. **(D)** Survival was monitored for 60 days after BM transplantation. Results are expressed as mean ± SEM (n≥5/group). *p≤0.05, **p≤0.01.

Colitis was induced by the administration of DSS (molecular weight, 40 kilodaltons; Sigma Aldrich). 2.5% DSS was dissolved in sterile, distilled water and administered *ad libitum* for 7 days. Fresh DSS solutions were prepared daily before use. Mice were sacrificed at day 8 following colitis induction (21, 28). Control animals were untreated. In survival experiments, animals were monitored daily for behavior, aspect alteration and body weight loss for a period of 21 days. Animals presenting signs of suffering (weight loss >20%, prostration, tremors) were immediately euthanized. The entire colon was removed from the caecum to the anus, then measured. Anti-RGMB (9D1) or isotype control antibody was given at indicated time points (**Figure 1C**).

### T cell proliferation *in vivo*

To assess the impact of anti-RGMb antibody on T cell proliferation, mice were injected with anti-RGMb antibody (9D1 or 2C9, 20 mg/Kg; i.p.). Following the first injection, they received 5x10^6^ bone marrow and 1x10^6^ luc^+^ T cells by i.v. route. Survival and representative bioluminescence images of luc^+^ T cell biodistribution was analyzed by Xenogen IVIS 100 *in vivo* imaging system.

### Cell isolation and flow cytometry

T cells were prepared from C57BL/6 splenocytes and lymph nodes by positive selection with anti-CD4 and anti-CD8 MicroBeads (Miltenyi Biotec). TCD-BM cells were prepared by flushing murine tibiae and femora with PBS supplemented with 2% FCS followed by depleting T cells with anti-CD4 and anti-CD8 MicroBeads (Miltenyi Biotec) reaching a purity >99%.

Mouse splenocytes or single small intestine cells digested with collagenase IA (Sigma-Aldrich) were washed twice in a staining buffer consisting of PBS supplemented with 1% fetal bovine serum. Flow cytometry followed routine procedures using 1x10^5^ cells stained per sample. Cells were labeled with CD4 [RM4-5], CD8 [53-6.7], CD25 [M-A251], CD69 [H1.2F3], CTLA4 [UC10-4B9], PD1 [29F.1A12] antibody, (Biolegend, San Diego, CA) and Lag3 [C9B7W] (BD Biosciences) to measure expression. Flow-cytometric analysis was conducted on a FACS LSR II (Becton Dickinson) and analyzed using FlowJo analysis program v7.6.5. Gating strategies are shown in **Suppl. Figure 1.**

### Mixed lymphocyte reaction

CD11b^+^ cells were isolated from C57BL/6 mice and were incubated with FACS-purified naïve T cells from BALB/c mice (TCR^+^CD62L^high^CD44^-^). Mixed cells were cultured for 7 days in RPMI 1640 supplemented with 2 mmol/L L-glutamine, 1 mmol/L sodium pyruvate, 100 U/mL penicillin, 100 μg/mL streptomycin, and 10% FCS (Gibco®). The naïve T and CD11b^+^ cell ratio were 5:1. Cytokines profile in the supernatant were analyzed using mouse ultrasensitive cytokine magnetic 10-Plex panels (Invitrogen).

### Cytokine measurement

For quantitative cytokine measurement, mouse spleens were harvested on day 9 after BMT or DSS treatment and splenocytes (0.5x10^5^ cells/well) were stimulated ex vivo or not with anti-CD3/anti-CD28 Dynabeads (ThermoFisher) in 96 well plates. Culture supernatants were collected at 72 hours and stored at -20°C until analysis for cytokine concentrations by a multiplex assay (Luminex, Life Technologies Logan, UT) as per manufacturer’s recommendations.

### RNA extraction, quantitative real-time PCR and gene expression microarray

Total RNA was isolated from the small intestine of mice using the RNeasy mini kit (QIAGEN). First-strand cDNA synthesis was performed using the SuperScript II kit (Life Technologies) and amplified using the Bio-Rad qPCR System (Bio-Rad) using specific primers for mouse.

Gut tissue was collected into an RNA stabilization buffer (Ambion) and was cut into small pieces and homogenized before loading onto a QIA shredder spin column (Qiagen). Details regarding the qRT-PCR assay can be found in **Suppl. Table 1**. Gene expression microarray was performed and analyzed as described in **Supplementary Materials and Methods**.

The list of 969 differentially expressed genes (Fold change >3) was uploaded onto the website of Ingenuity Pathway Analysis. Each gene was mapped to its corresponding gene object signaling in the Ingenuity Pathways Knowledge Base and biological function categories of each gene were annotated by Ingenuity Pathway Analysis (Qiagen).

Details regarding real-time PCR primers and analysis can be found in **Supplementary Materials and Methods**.

### Western blotting

Mouse small intestines were lysed in RIPA lysis buffer (Invitrogen) containing protease inhibitor mixture (Roche) for 30 min on ice. After centrifugation for 10 min at 4°C, the supernatant was assayed for protein concentration by colorimetric assay (BCA kit, Pierce). The lysates were subjected to Western blotting analysis using anti-RGMb antibody (Abcam, ab-96727) and anti-β-actin antibodies (Santa Cruz, sc-47778) as indicated.

### Immunohistochemistry staining

Intestinal tissues were removed, the length measured and fixed in formalin. Five-micrometer sections were cut longitudinally, and slides stained with hematoxylin and eosin [H&E] using a standard protocol. Based on the existing literature, eight histological components were assessed: inflammatory infiltrate, goblet cell loss, hyperplasia, crypt density, muscle thickness, submucosal infiltration, ulcerations, and crypt abscesses (all categorized from 0–3 (29). A total histological severity score, ranging from 0 to 24, was obtained by summing the eight-item scores. GvHD model histopathology was scored as previously described (30).

### *In vivo* bioluminescence imaging

Bioluminescence imaging was performed as described previously (Xenogen) (31). Briefly, firefly luciferin (Biosynth) was injected intraperitoneally 10 min prior to image acquisition with an IVIS spectrum imaging system (Xenogen). Images were analyzed with Living Image Software 4.2 (Xenogen).

### Statistical analysis

GraphPad Prism statistics software (GraphPad Software Inc., San Diego, CA, USA) was used for analysis and data graphing. Results were analyzed using a non-parametric test (Mann Whitney tests), T-test expressed in terms of probability (*p*). Kaplan Meier analysis, Long-rank (Mantel-Cox) test were used for survival curves comparisons. Differences were considered significant when *p*<0.05. All data are expressed as mean ± SEM.

## Results

### Total body irradiation increases RGMb expression in mouse intestine tissue

RGMb expression was significantly increased in the large and small intestine at 24h after total body irradiation (TBI, without HCT), as assessed by mRNA expression (**Figure 1A**) and at the protein level (**Figure 1B**). BMPR1a, a co-receptor of the RGMb protein complex, was also significantly increased in the gut at 6h and 24h after TBI (**Figure 1A**). We did not observe a significant difference in RGMb or BMPR1a expression in the spleen after TBI (**Figure 1A**).

### Anti-RGMb blocking monoclonal antibody 9D1 protects against GvHD

The RGMb mAb 9D1 blocks both RGMb-PD-L2 and RGMb-BMP-2/4 interactions. In contrast, the PD-L2 mAb 2C9 blocks RGMb-PD-L2 but not PD-1-PD-L2 interactions (6). We tested these antibodies in an established acute GvHD mouse model by transplanting C57BL/6 donor T cell depleted bone marrow (TCD-BM) supplemented with C57BL/6 CD4^+^/CD8^+^ T cells into recipient BALB/c mice (32). MAbs 9D1 or 2C9 were administered i.p. into the recipient mice at the indicated time points before and after allogenic bone marrow transplantation (BMT) (**Figure 1C**). Treatment with 9D1 but not the 2C9 or isotype control antibodies protected the recipient mice against GvHD with improved survival (75% versus 30% survival rate at 60 days after transplantation, p<0.05) (**Figure 1D**).

### Anti-RGMb blocking monoclonal antibody 9D1 reduces T cell infiltration into the intestine

**Figure 2:**
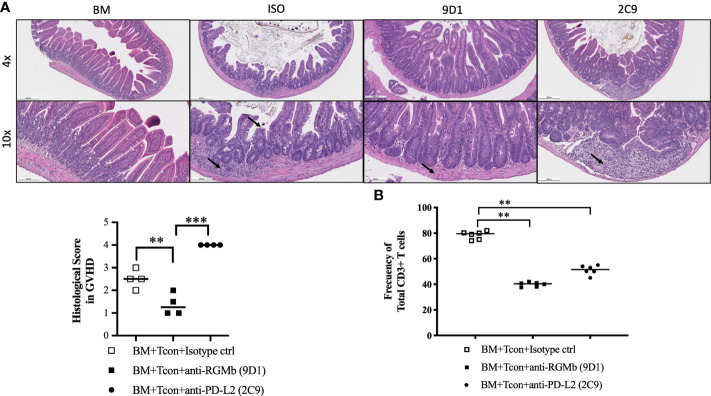
Treatment with anti-RGMb antibody 9D1 reduces GvHD. **(A)** Large intestine histopathology was assessed by H&E staining on normal, isotype control, 9D1- and 2C9-antibody-treated mice (20 mg/Kg; i.p.) at 9 days after BM transplantation. Histology is shown at 4x (scale bar 500 μm) and 10x (scale bar 200 μm) and histology score in GVHD is shown. Small arrows represent inflammatory infiltrate and crypt structure. **(B)** CD3^+^ T cell numbers of gut CD45^+^ cells were calculated by flow cytometry. Results are expressed as mean ± SEM (n≥5/group). **p≤0.01, ***p≤0.001, One-way ANOVA comparisons.

To further confirm that anti-RGMb mAb blockade of the BMP-2/4 and/or NEO1 binding site reduced GvHD, the recipient mice were sacrificed at 9 days after transplantation, and intestinal tissues were harvested. Histopathological analysis of mouse intestine showed that treatment with 9D1 markedly decreased immune destruction of small and large intestinal villi and ameliorated the GvHD histology score, in the intestine of 9D1-treated mice compared to control mice, measured by a pathologist blinded to the treatment groups (*p*<0.005, **Figure 2A**).

Tissues from the small intestine of recipient mice were digested into single cell suspensions, and flow cytometry was used to quantify T cell infiltrates. A decreased frequency of CD3^+^ T cells was observed in the gut of GvHD mice treated with 9D1 mAb compared to isotype control-treated mice (*p*<0.005, **Figure 2B**).

To profile the T cell phenotype, we obtained splenocytes 9 days after BMT transplantation and measured cytokine production by ex vivo CD3-CD28 bead (GibcoTM) activation (**Suppl. Figure 3**). Levels of VEGF were significantly increased after 9D1 treatment compared to the isotype control antibody treated mice (p ≤ 0.05). Levels of IL-5, IL-13, IL-17, GM-CSF and IFN-γ decreased after 9D1 treatment. No significant differences were observed in IL-1β, IL-2, IL-4, IL-6, IL-12, MIG, MIP1a, TNF-α and MCP1 levels after 9D1 treatment compared to controls.

### Anti-RGMb mAb treatment alters gut immune gene expression in GvHD

RGMb blockade reduces inflammation in the intestine tissue. Cluster analysis generated from DNA microarray data of gene expression values in the gut 9 days after BMT (**Suppl. Figure 2**) showed an increase in gene expression profiles associated with immune cell trafficking, tissue development and function, and cell-to-cell signalling and interaction following 9D1 or isotype control treatment. SAA1, TLR2, TLR4, BMP4 are relevant examples of genes upregulated following 9D1 treatment, 9 days after BMT. CD34 expression is also upregulated showing the role of RGMb in cells development. Myoferlin (MYOF) gene is upregulated, a calcium/phospholipid-binding protein that plays a role in the plasmalemma repair mechanism of endothelial cells. It is involved in endocytic recycling and it is implicated in VEGF signal transduction by regulating the levels of the kinase insert domain receptor (KDR). The expression CD36 and TIMP2 is also shown to be affected. These markers are involved in tumor metastasis.

### Anti-RGMb mAb 9D1 does not alter graft-versus-tumor effects

**Figure 3:**
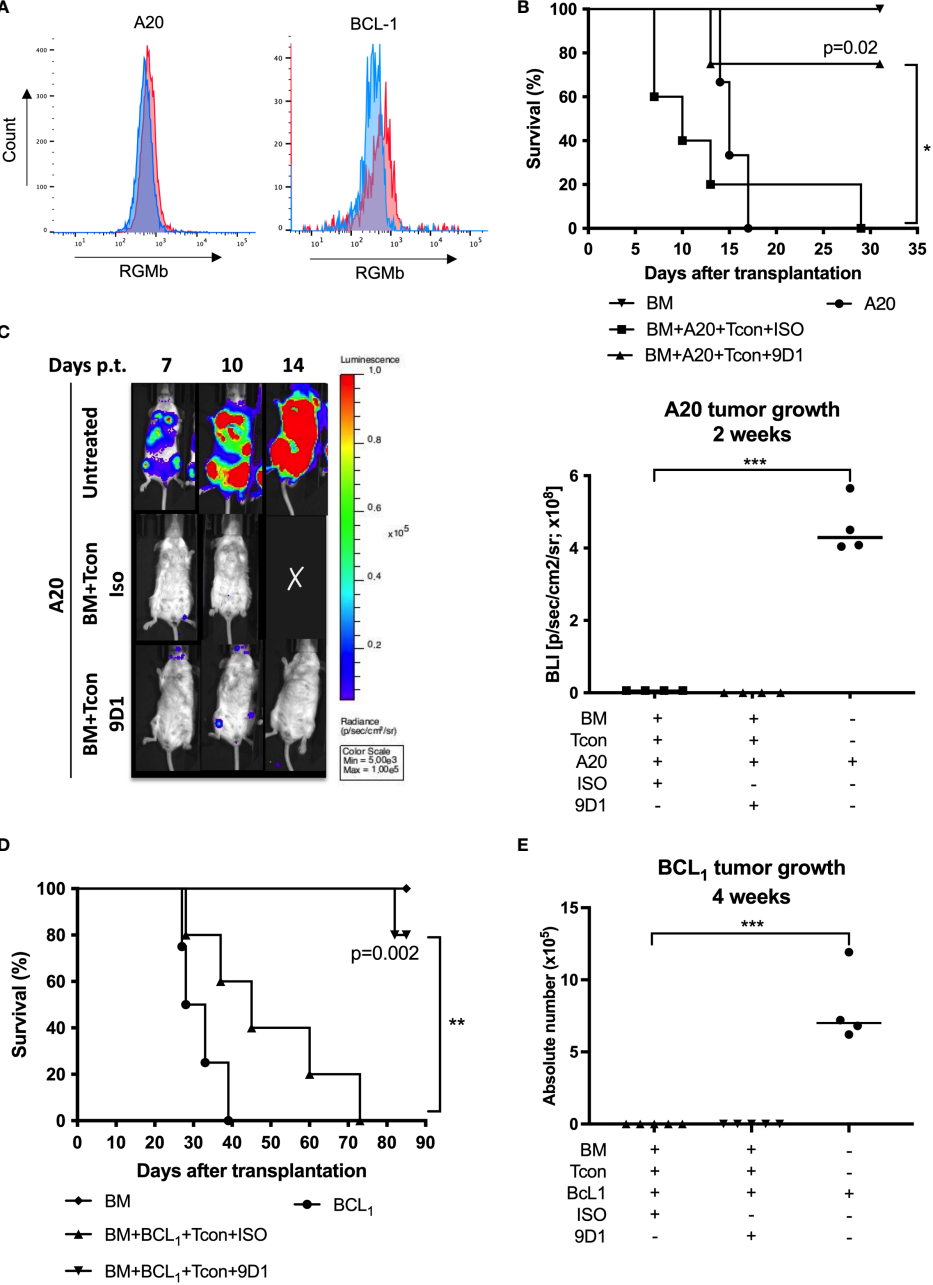
Anti-RGMb antibody 9D1 therapy enhanced survival in graft-versus-tumor model. **(A)** Overlay of a negative population (Blue) onto the stained population (Red) allows easy identification of the RGMb expression on A20 or BCL1 cells. **(B)** Mice survival was monitored for 31 days after A20 transplantation on isotype control and 9D1-antibody-treated mice (20 mg/Kg; i.p.). **(C)** Representative images and quantification of signals were done using a living imaging program. Results are expressed as mean ± SEM (n≥4/group). **(D)** Mice survival was monitored for 80 days after BCL1 transplantation. **(E)** Tumor growth was monitored by flow cytometry 4 weeks after BCL1 transplantation. *p≤0.05, **p≤0.01, ***p≤0.001, One-way ANOVA comparisons.

We further examined if 9D1 treatment could reduce GvHD without interfering with the therapeutic GVT effect mediated by BMT. To test this hypothesis, mouse B cell lymphoma cells (A20) carrying luciferase reporter gene or B-cell leukemia lymphoma (BCL_1_) cells without luciferase reporter gene were transplanted with allogeneic TCD-BM and T cells, and the recipient mice received 3 injections of 9D1 or isotype control mAbs (400 µg/mouse, i.p.). RGMb expression on A20 and BCL1 cells was measured by flow cytometry. Tumor cells did not show cell surface expression of RGMb (**Figure 3A**). Recipient wild type mice, conditioned with TBI, all died from leukemia at day 17 or 21 after inoculation of A20 or BCL_1_ cells, respectively. Transplantation of TCD-BM and donor T cells plus anti-RGMb treatment, but not isotype control, significantly improved the survival rate in the recipient mice (**Figures 3B, D**). By tracking the lymphoma with luciferase signaling *in vivo* for A20 or cell counts for BCL1, we found that allogeneic BMT mediated the clearance of inoculated A20 and BCL_1_ cells in both anti-RGMb or isotype control treated mice (**Figures 3C, E**). Anti-RGMb therapy was able to reduce GvHD, without interfering with the beneficial GVT effect mediated by allogeneic BMT, enabling prolonged survival of the mice. In contrast, isotype control-treated mice cleared the leukemia but the mice died from GvHD.

### RGMb mAb treatment protects against human xenograft GvHD in NSG mouse recipients

**Figure 4:**
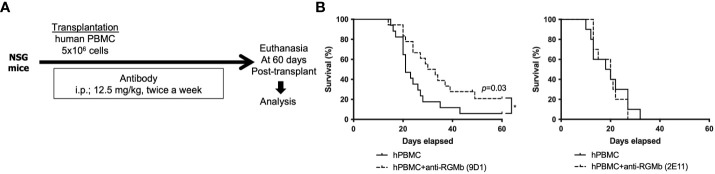
Anti-RGMb antibody therapy with 9D1 but not 2E11 ameliorates xenograft GvHD in mice. **(A)** To assess the impact of anti-RGMb antibody treatment in T cell xenoresponse, female NSG mice were irradiated with 250 cGy and subsequently injected with 5x10^6^ human PBMCs. On days -1, 3, 7 and 11 after PBMC transfer the RGMb antibody was injected i.p. (12.5 mg/kg). **(B)** Survival was monitored until 60 days after transplantation. Three independent experiments were done. Results are expressed as mean ± SEM (n=5/group). *p≤0.05.

We next asked if RGMb blockade could ameliorate xenograft GvHD in irradiated NSG recipient mice injected i.v. with human peripheral blood mononuclear cells (PBMCs) (5x10^6^/mouse). Anti-RGMb mAbs 9D1 and 2E11 have similar blocking characteristics, but 9D1 has high affinity for both mouse and human RGMb while 2E11 binds only to human RGMb. Human BMP-2/4 are reported to bind to murine RGMb (6). We hypothesized that if mAb 9D1 but not 2E11 was effective at preventing xenograft GvHD then RGMb blockade-mediated protection from xenograft GvHD is dependent on recipient mouse epithelium or antigen presenting cells, while if mAb 2E11 was effective at preventing xenograft GvHD, then RGMb blockade-mediated protection from xenograft GvHD is acting primarily through RGMb on donor mononuclear cells (human). Both the 9D1 and 2E11 antibodies were administrated on days -1, 3, 7, 11 after administration of human PBMCs and GvHD symptoms and survival were monitored after transplantation. Treatment with 9D1 but not 2E11 RGMb mAb significantly reduced GvHD scores and improved survival (**Figure 4**), suggesting that RGMb blockade-mediated protection from xenograft GvHD is dependent on mouse epithelium or antigen presenting cells in the recipient.

### Anti-RGMb blocking monoclonal antibody 9D1 prevents IBD in mice

**Figure 5:**
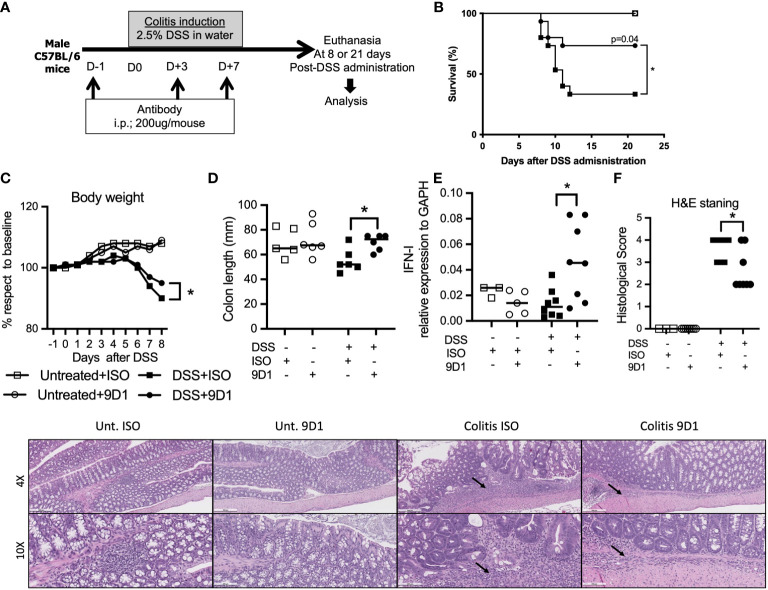
Treatment with anti-RGMb antibody 9D1 prevents inflammatory bowel disease. **(A)** To assess the capacity of anti-RGMb antibody treatment to prevent colitis, mice were injected with anti-RGMb mAb 9D1 or Isotype control antibody (12.5 mg/Kg; i.p.). 24h after first injection mice were treated or not with 2.5% DSS in their drinking water for 7 days to induce colitis. Mice were euthanized at day 8 or 21. **(B)** Mice survival was monitored for 21 days after DSS administration. **(C)** Mice body weight changes during the induction of colitis. Statistical significance of the weight changes on day 8 was determined using the Student’s test. **(D)** Gross morphological changes and **(E)** IFN-I expression in gut of the colon on day 8 after 7 days of 2.5% DSS and 1-day drinking water. **(F)** Gut histopathology was performed by H&E staining on controls versus control isotype antibody- and 9D1-treated mice at 8 days after DSS administration (n=8/group). Histology is shown at 4x (scale bar 500 μm) and 10x (scale bar 200 μm). Small arrows represent inflammatory infiltrate. Results are expressed as mean ± SEM (n≥3/group). *p≤0.05. One-way ANOVA comparisons. .

The RGMb mAb 9D1 blocks RGMb-PD-L2 and RGMb-BMP-2/4 interactions (6). In order to understand how broadly RGMb might be involved in gut mucosal immunity, we assessed the impact of the BMP signaling pathway modulation in an inflammatory bowel disease (IBD) mouse model. C57BL/6 mice were injected with three doses of either 9D1 or isotype control antibody (200 µg/mouse; i.p.). Beginning one day after the first administration, mice were given 2.5% DSS through their drinking water for 7 days to induce colitis (**Figure 5A**). Mortality was monitored over 21 days. 9D1 therapy protected the DSS treated mice against weight loss and death due to colitis, whereas control isotype antibody therapy failed to protect the recipient mice (**Figures 5B, C**). As shown in **Figure 5D**, the colon lengths were similar among untreated animals, but DSS treatment led to a significant reduction in colon length in isotype control antibody treated mice, but not in 9D1 treated mice. 9D1 treatment significantly increased Type I IFN, as measured by quantitative PCR (**Figure 5E**). Histopathological analysis of the gut showed that 9D1 treatment in colitic mice significantly ameliorates inflammation, ulceration, and tissue remodeling, compared to isotype control antibody treated mice. This was confirmed by the histopathological score evaluating both inflammation and gut tissue remodeling (3.7 ± 0.5 vs 2.7 ± 0.9 in isotype control and 9D1 DSS-treated mice) (**Figure 5F**). These resuts show the significance of RGMb signaling in gut homeostasis.

To profile the T cell phenotype, we obtained splenocytes 9 days after DSS treatment and measured cytokine production by ex vivo CD3-CD28 bead (GibcoTM) activation (**Suppl. Figure 4**). 9D1 treatment decreased IFN-γ and increased IL-10 compared to the isotype control antibody treated mice (p ≤ 0.05). No significant differences were observed in IL-1β, IL-2, IL-4, IL-6, IL-9, IL-12p70, IL-13, IL-17, IL-18, IL-22, TNF-α, GM-CSF, IFN-γ levels after 9D1 treatment compared to controls. Levels of IL-5, IL-10 were significantly increased after 9D1 treatment.

Treatment with 9D1 antibody have ameliorated cytokines production after a mixed lymphocyte reaction between CD11b^+^ cells and naïve T cells (**Suppl. Figure 5**). No differences were observed in IL-1β levels compared to control. Levels of IL-2, IL-4, IL-5, IL-6, IL-9, IL-10, IL-12, IL-13, IL-18, TNF-α, GM-CSF and IFN-γ significantly decreased after 9D1 treatment.

## Discussion

RGMb was originally described as a key factor in the early developing central nervous system, controlling neuronal cell differentiation, migration, and apoptosis (5), but plays a limited role in adults. RGMb expression has now been identified in several other tissues and organs, including bone, lung, gut (33), and the immune system (5, 6, 34). In mouse gut tissue, RGMa and RGMb, but not RGMc, was detected in enteric ganglia cells and intestinal epithelium, predominantly in the proliferative crypt compartment (33). Given the expression of RGMb in the gut and recent evidence for the importance of this signaling hub in lung and gut mucosal immunity (12, 23). We hypothesized that RGMb is involved in immune responses within the gut mucosa.

The intestine and the intestinal crypt in particular are a primary target of T-cell driven inflammation in GVHD (35, 33). It is known that the damage to the gut induced by radiation and/or chemotherapy conditioning in HCT results in the production of inflammatory chemokines and cytokines which can contribute to the development of pathologic donor T cells that cause acute GVHD. We found an increase in RGMb expression with TBI conditioning-associated damage to the gut suggesting that this pathway might contribute to GVHD.

The crystal structure of RGMb shows that neogenin and BMP-2/4 bind in a bridging structure which leads to the recruitment of BMPRI and other receptors. This interaction can be blocked by specific mAbs such as 9D1 (6) or 2E11. In mice, PD-L2 binds to a distinct region and this interaction can be blocked by specific mAbs such as 2C9 or 9D1. One major finding of our study is that blocking the neogenin and BMP-2/4 binding site on RGMb results in decreased infiltration of T cells into the gut in GvHD mouse disease models. Blockade of the neogenin and BMP-2/4 binding site with 9D1 reduced small and large intestine immune destruction in GvHD and improved survival in both mouse allogeneic HCT and xenograft models. In contrast, blockade of the RGMb binding site on PD-L2 exacerbates GvHD in mouse models of allogeneic HCT proliferative effect. RGMb–PD-L2 interaction occurs in both mice and humans (6). The pathway consisting of the programmed cell death 1 (PD-1) receptor (CD279) and its ligands PD-L1 (CD274) and PD-L2 plays an important role in the induction and maintenance of peripheral tolerance and for the maintenance of the stability and the integrity of T cells (36). This suggests an important and wider role for RGMb in gut mucosal immunity than previously known.

Since BMP2, BMP4, and neogenin can be expressed by T cells and have been shown to regulate the activation of naïve T cells (37), it is possible that RGMb may regulate T cell proliferative responses by interaction of these molecules with PD-L2 expressed on antigen-presenting cells or other cells.

Although it remains unclear when and how T cells in these models make neogenin or BMP-2/4, previous studies have shown that CD4^+^ and CD8^+^ T cells can express neogenin when activated and that human naïve T cells can express BMP-2/4, capable of autocrine signalling (37).

Moreover, RGMb have been described as a novel binding partner of CTLA-4. RGMb expression was detected at high levels in dendritic cell subsets that have been suggested to have tolerogenic capabilities. RGMb binds an extracellular domain of CTLA-4, and specifically strengthens the binding of the monomeric, soluble form of CTLA-4 (sCTLA-4) to CD80, enhancing CTLA-4’s suppressive effect on co-stimulation (18). The findings advance our understanding of CTLA-4 activity, as well as identify the RGMb/CTLA-4 binding interface as a potential target for the development of novel immune checkpoint blockade therapies.

In the allogeneic HCT setting, the conversion of naive T cells to effector cells, their proliferation, metabolism are thought to be the principal sources of alloreactive T cells which drive the pathophysiology of the disease (38, 39). On day 9 post BMT, we observed a decreased frequency of CD3^+^ T cells in the gut of GvHD mice treated with 9D1 mAb compared to isotype control-treated mice. In addition, microarray analysis showed differential expression of genes between treated and untreated groups. More specifically, genes associated with immune cell trafficking, tissue development and function, and cell-to-cell signalling, and interaction were upregulated following 9D1 treatment. SAA1, TLR2, BMP4 are relevant examples of genes upregulated following 9D1 treatment, 9 days after BMT. Interestingly serum amyloid A1 (Saa1) has been associated with a protective role in inflammatory bowel disease (40) and is upregulated in the 9D1 treated animals. This may explain why blockade of the RGMb: neogenin/BMP2,4 interaction was successful in ameliorating disease, while preserving the GVT effect in the 9D1 treated animals.

In the IBD setting, the conversion of naive T cells to effector cells is thought to be the principal source of auto-reactive (in DSS) T cells that drive the pathophysiology of the disease. It remains unclear exactly how the inflammation in the gut of mice with colitis is reduced by RGMb blockade; however, we observed that in DSS-treated mice, the colon length was reduced in the isotype and not the 9D1 treated animals. In addition, Type I IFN expression was significantly increased in the gut on day 8 after DSS treatment. Baseline constitutive expression of IFN-I is very low in the intestines, typical of most tissues (41–45). Type I interferons can function as a key factor in the attenuation of an active immune response and therefore to dampen inflammation. IFN-I inhibits the expression of IL-8, a chemotactic cytokine responsible for recruiting neutrophils and leukocytes to areas of inflammation (46, 47) and of IL-17, *via* inhibition of Th17 differentiation (48, 49). IFN-I antagonize the effects of local IL-17 by down-regulating the expression of IL-1β, IL-23, and osteopontin, and by inducing the production of the anti-inflammatory cytokine IL-27 in DCs (49, 50). This may explain why blockade of the RGMb: neogenin/BMP2,4 interaction was successful at ameliorating gut inflammation.

In conclusion, our data demonstrate a previously unknown role of RGMb in T cell function, as well as in gut mucosal immunity, in allogeneic HCT disease and IBD models. The therapeutic targeting of this pathway in allogeneic HCT may offer a new strategy to prevent or treat GvHD without impairing GVT. Likewise, the involvement of this pathway in gut mucosal immunity and potentially IBD may be an important therapeutic target in early onset or refractory cases not responsive to traditional therapeutic intervention targeting other established inflammatory pathways.

## Data availability statement

The original contributions presented in the study are included in the article/**Supplementary Material**. Further inquiries can be directed to the corresponding authors.

## Ethics statement

The animal study was reviewed and approved by IACUC.

## Author contributions

MP-C designed and performed research, analyzed data and interpretation. MP-C and BI wrote and edit the manuscript. BI, KH, H-HW, TE, KS, S-WT, CB, and BX performed research. GF and RD provided RGMb reagents, insight into the RGMb pathway, and overall feedback, EM is the senior author of this study and provided overall guidance. NK evaluated histopathology and took photomicrographs. All authors contributed to the article and approved the submitted version.

## Funding

This work was supported by PO1AI056299, P50 CA101942 and P50 CA206963 (GJF), National Institutes of Health National Heart, Ling, and Blood Institute 1K08HL119590 Meyer, and National Institutes of Health.

## Acknowledgments

The authors thank Kent P. Jensen, Jeanette Baker and Suparna Dutt for technical assistance.

## Conflict of interest

EM, RD, and GF are co-founders and equity holders in Triursus Therapeutics. EM is a co-founder of GigaGen, Inc. GF has patents/pending royalties on the PD-1/PD-L1 pathway from Roche, Merck MSD, Bristol-Myers-Squibb, Merck KGA, Boehringer-Ingelheim, AstraZeneca, Dako, Leica, Mayo Clinic, and Novartis. GF has served on advisory boards for Roche, Bristol-Myers-Squibb, Xios, Origimed, Triursus, iTeos, NextPoint, IgM, Jubilant, GV20, Geode, IOME, and Invaria. GF has equity in Nextpoint, Xios, iTeos, IgM, GV20, Trillium, Invaria, and is a co-founder and equity holder in Triursus.

The remaining authors declare that the research was conducted in the absence of any commercial or financial relationships that could be construed as a potential conflict of interest.

## Publisher’s note

All claims expressed in this article are solely those of the authors and do not necessarily represent those of their affiliated organizations, or those of the publisher, the editors and the reviewers. Any product that may be evaluated in this article, or claim that may be made by its manufacturer, is not guaranteed or endorsed by the publisher.

## Abbreviations

RGMb; Repulsive Guidance Molecule b, GvHD; Graft-versus-host disease, HCT; Hematopoietic cell transplantation, GVT; Graft-versus-tumor, IBD; Inflammatory bowel disease, BMPs; Bone morphogenetic proteins, TBI; Total body irradiation, DSS; Dextran sulphate sodium.
