# Supplementary material for: Immunoregulatory effects of RGMb in gut inflammation
Source: Front Immunol. 2022 Nov 7;13:960329. doi: 10.3389/fimmu.2022.960329 (PMC9676481; doi:10.3389/fimmu.2022.960329)
Supplement: Supplementary file 1 [file DataSheet_1.docx]

**Supplementary information**

**Immunoregulatory effects of RGMb in gut inflammation.**

**Magdiel Pérez-Cruz^1*^†, Bettina Iliopoulou^1*^†, Katie Hsu^1^, Hsin-Hsu Wu^1^, Tom Erkers^1^, Kavya Swaminathan^1^, Sai-Wen Tang^1^, Cameron S. Bader^1^, Neeraja Kambham^2^, Bryan Xie^1^, Rosemarie H. Dekruyff^3^, Gordon J. Freeman^4^ and Everett Meyer^1*^**

1 Division of Blood and Marrow Transplantation, Department of Medicine, Stanford University School of Medicine, Stanford, California, United States of America.

2 Department of Developmental biology, Stanford University School of Medicine, Stanford, California, United States of America.

3 Department of Pediatrics, Stanford University School of Medicine, Stanford, California, United States of America.

4 Department of Medical Oncology, Dana-Farber Cancer Institute, Harvard Medical School, Boston, MA 02215.

† These authors have contributed equally to this work and share first authorship

Running Title: Immunoregulatory effects of RGMb in gut.

* Correspondence:

Everett H. Meyer, MD, PhD

Division of Blood and Marrow Transplantation, Department of Medicine, Stanford University School of Medicine, 300 Pasteur Drive, Room H3249, Stanford, CA 94305-5623, United States of America.

Phone: 650 725 5816

Fax: 650 497 3603

E-mail: evmeyer@stanford.edu

Conflict of Interest Statement

E.H.M., R.H.D, and G.J.F are co-founders and equity holders in Triursus Therapeutics. E.H.M is a co-founder of GigaGen, Inc. GJF has patents/pending royalties on the PD-1/PD-L1 pathway from Roche, Merck MSD, Bristol-Myers-Squibb, Merck KGA, Boehringer-Ingelheim, AstraZeneca, Dako, Leica, Mayo Clinic, and Novartis. GJF has served on advisory boards for Roche, Bristol-Myers-Squibb, Xios, Origimed, Triursus, iTeos, NextPoint, IgM, Jubilant and GV20. GJF has equity in Nextpoint, Xios, iTeos, IgM, GV20 and is a co-founder and equity holder in Triursus.

**Material and methods (supplementary information)**

**Microarrays**

Total RNA was extracted, using a RNeasy Micro Kit (Qiagen) and its quality was assessed by an Agilent 2100 Bioanalyzer. Total RNA (from 500 pg - 2 ng) was reverse transcribed into cDNA followed by in vitro transcription use GeneChip™ WT Pico (Affymetrix, Santa Clara, CA). Labeled cDNAs were hybridized to (Affymetrix) according to the manufacturer’s protocol, and the chips were scanned using a GeneChip Scanner 3000 GeneChip™ Mouse Gene 2.0ST- this is a whole transcript design with probes at 3' as well as exon. Catalog number: 902118 (Affymetrix). Background correction, normalization and estimation of gene expression was performed using the Robust Multiarray Average (RMA) method in R/Bioconductor ([www.bioconductor.org](http://www.bioconductor.org/)). Following aggregation, the samples were re-normalized using quantile normalization.

Gene expression microarray was performed from gut as previously described (Jiang et al., 2015); (Lin et al., 2016); (Li et al., 2018). Briefly, Cy3-labeled cDNA for microarray hybridization was prepared with the One-Color Low Input Quick Amp Labeling Kit (Agilent, USA) and then purified using the RNeasy Mini Kit (Qiagen, Germany). Cy3-labeled cDNA was fragmented and hybridized to the array for 17 h at 65 °C in a rotating Agilent hybridization oven. After hybridization, arrays were washed and dried, then scanned immediately on the Agilent Microarray Scanner. Intensity values of each scanned slide were extracted using Agilent Feature Extraction software (version 10.7.3.1; Agilent Technologies).

Raw data analyses were performed with GeneSpring GX software (Version 12.0; Agilent Technologies). The intensity values were log 2 transformed by quantile normalization (Bolstad et al., 2003). The Welch*-*test (*p-*values) was applied to identify differentially expressed genes in NOA compared to OA. The *p*-values were corrected by the false discovery rate of Benjamini and Hochberg (*q* values) analyses. Fold change (FC) values were calculated for each gene as the difference between the mean intensity of the NOA samples and the mean intensity of the OA samples. Genes with an FC value >2 or <1/2 and a *q-*value <0.05 were considered to be differentially expressed. Quality control analysis of microarray gene expression data was performed as previously described (Zahurak et al., 2007).

**Primers.**

Quantitative RT-PCR was performed to quantify mRNA of interest (Supplementary Figure 1). Results were expressed as mean ± SEM of the relative gene expression calculated for each experiment in folds (2^-ΔΔCt^) using GAPDH as a reference.

**Isolation of Murine Mesenchymal Stem Cells**

The method of isolating mouse bone marrow was described previously (Lin et al., 2015). In brief, bone marrow was collected from the femurs and tibias of C57BL/6J male mice aged 8 weeks (three mice). Institutional guidelines for the care and use of laboratory animals were observed in all aspects of this project. The cells were carefully suspended and passed through a 70 μm strainer, spun down, and resuspended in α‐MEM supplemented with 10% MSC certified fetal bovine serum (FBS, Invitrogen) and antibiotic antimycotic solution (100 U of penicillin, 100 μg of streptomycin, and 0.25 μg of Amphotericin B/ml; Hyclone, Thermo Scientific). The media was replaced the next day with fresh media to remove the unattached cells (passage 1). The cells were allowed to grow to confluence for 2 weeks, and then isolated by murine mesenchymal stem cell enrichment kit (STEMCELL Technologies). The cellular morphology was observed under a microscope (Axio Observer 3.1, Zeiss, Oberkochen, Germany). The immunophenotypes of isolated MSCs (Sca1^+^/CD90.2^+^/CD105^+^/CD44^+^/CD45^−^/CD34^−^) were characterized by LSR II flow cytometer (BD Bioscience) as described previously (Lin et al., 2015). The data were analyzed by Flowjo X 10.0 (Tree Star Inc.). The experiments were confirmed twice independently.

For macrophage isolation, the bone marrow cells were washed 3 times with culture medium [Roswell Park Memorial Institute (RPMI) 1640 medium, supplemented with 10% heat inactivated FBS, and the antibiotic-antimycotic solution], resuspended in the culture medium containing 30% of L929 cell conditioned medium and 10 ng/ml mouse macrophage colony stimulating factor (R&D Systems, Minneapolis, MN, USA), and replated in T-175 culture flasks at a concentration of 4 × 10^7^ cells per flask. Cells were allowed to expand for 5–7 d, with a medium change at the second day to remove nonadherent cells.

The trained or control MSCs were treated with 20 ng/ml IFN-γ plus 20 ng/ml TNF-α or 1 μg/ml LPS (5) for 7 h at 3 or 7 d later. Cellular RNAs were extracted by using RNeasy RNA Purification Kit (Qiagen, Venlo, The Netherlands). RNAs were reverse transcribed into cDNA using a high-capacity cDNA archive kit (Thermo Fisher Scientific).

**Suppl. Figure 1.** **Gating strategy for isolation and analysis of mouse naïve CD8^+^ T cells from the spleen and lymph nodes.** Naïve CD8^+^ T population was first enriched by depletion of magnetically labeled non-target cells using an isolation kit from Miltenyi Biotec, USA. Cells were incubated with different antibodies to identify naïve CD8^+^ T subset. Live cells (gate A) were gated based on forward and side scatter. Not-single cells or Dead cells were excluded from further analysis (gate B and gate C, respectively). The CD45^+^ (gate D) and CD3^+^ (gate E) cells were selected for further analysis and examined for the expression of CD8 and CD4 (gate F). CD44^-^ CD62L^+^ (gate G) population represents the naïve T CD8^+^ and were further analyzed.

**Suppl. Table 1.** Primers for Real-time PCR of mouse samples.

| **Mouse Gene** | **Forward primer** | **Reverse primer** |
| --- | --- | --- |
| GAPDH | TTGCTGTTGAAGTCTCAGGAG | TGTGTCCGTCGTGGATCTGA |
| RGMb | TGCCGCTGATGGGTTTGAC | GCTGTGGTAGTTACAGGGGTC |
| Neogenin | TTGCTCGGCATATTCTGAGCC | TGGCGTCGATCATCTGATTCTAA |
| BMPRIa | TGGCACTGGTATGAAATCAGAC | CAAGGTATCCTCTGGTGCTAAAG |
| BMPRIb | CCTCGGCCCAAGATCCTAC | CCTAGACATCCAGAGGTGACA |
| BMP2 | GGGACCCGCTGTCTTCTAGT | TCAACTCAAATTCGCTGAGGAC |
| BMP4 | ATTCCTGGTAACCGAATGCTG | CCGGTCTCAGGTATCAAACTAGC |
| IL-4 | GGTCTCAACCCCCAGCTAGT | GCCGATGATCTCTCTCAAGTGAT |
| IL-10 | CTTACTGACTGGCATGAGGATCA | GCAGCTCTAGGAGCATGTGG |
| IL-17 | TCAGCGTGTCCAAACACTGAG | CGCCAAGGGAGTTAAAGACTT |
| IFN-γ | GCCACGGCACAGTCATTGA | TGCTGATGGCCTGATTGTCTT |
| IFN-I | TGGATGCCCAGCAGATCAAG | CCATGCAGCAGATGAGTCCT |
| CD34 | GGTAGCTCTCTGCCTGATGAG | TGGTAGGAACTGATGGGGATATT |
| CD109 | TCCCGCTTTCTGGTGACAG | ACCTGAGCCTTTACAAGGACC |
| CD274 | GCTCCAAAGGACTTGTACGTG | TGATCTGAAGGGCAGCATTTC |
| MerTk | CAGGGCCTTTACCAGGGAGA | TGTGTGCTGGATGTGATGTTC |
| Foxq1 | AAATTGGAGGTGTTCGTCCCA | TCCCCGTCTGAGCCTAAGG |
| Tm4SF4 | AAGCCACCTTTCGGATGAGG | CGCAGCAGTCGTTGTTCTG |
| SAA1 | CCAGGAGACACCAGGATGAA | TCATGTCAGTGTAGGCTCGC |


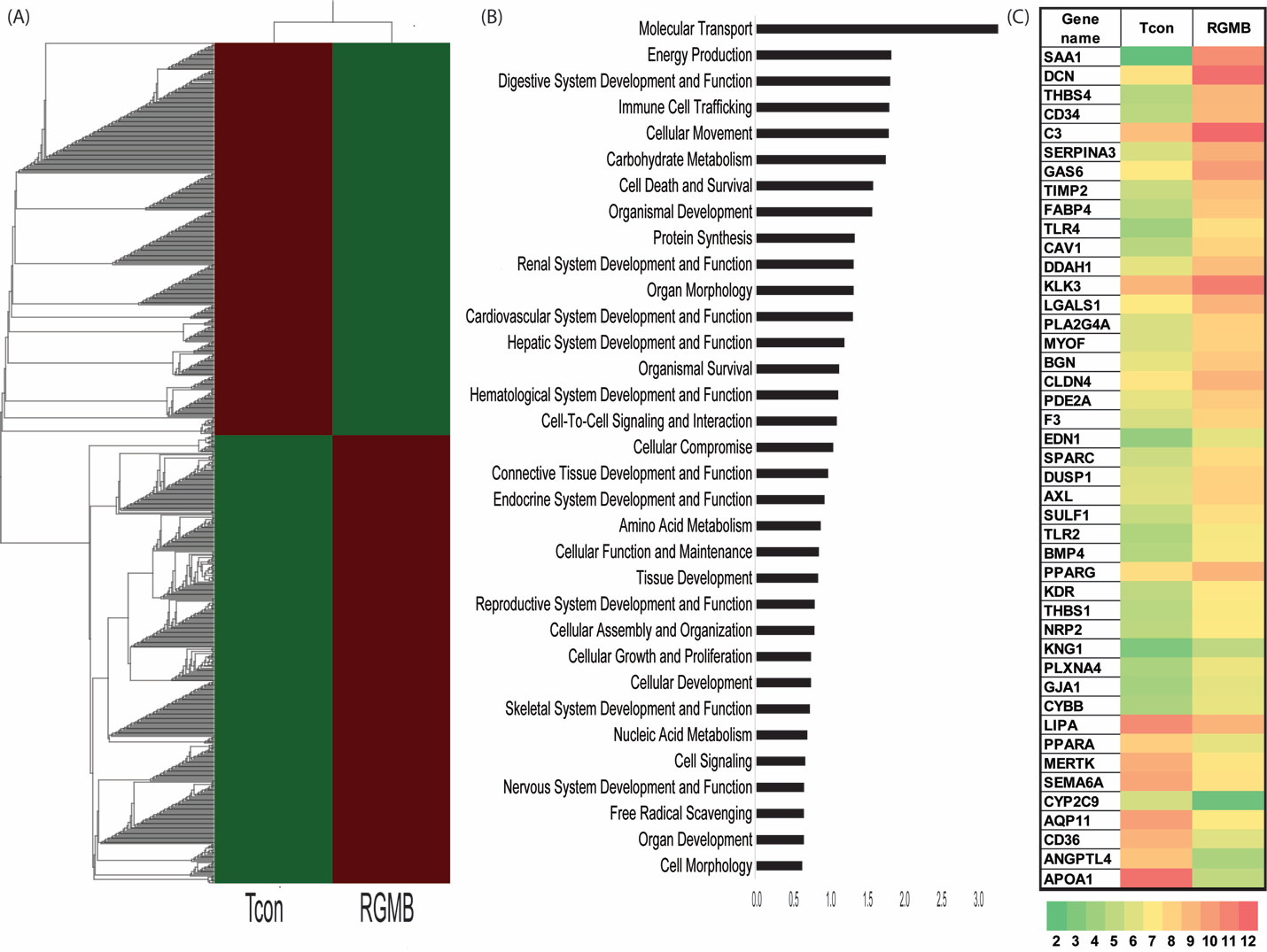


**Suppl. Figure 2. Cluster analysis. (A)** Cluster of gene expression in small intestine, 9 day after treatment with anti-RGMb antibody (9D1) or isotype control in GvHD in vivo. Fold change was calculated by RGMb versus T Bi-weight Avg Signal (Log2). **(B)** Pathways and **(C)** Relevant genes after anti-RGMb antibody (9D1) treatment. 2: Low and 12: High.

**Suppl. Figure 3. Treatment with 9D1 antibody promote anti-inflammatory response in GvHD model.** Protein levels was measured by multiplex assay in the supernatants after 3 days of exvivo activation with anti-CD3/CD28 beads at 1:2 ratio. Results are expressed as mean ± SEM (n≥3). **p<0.01 compared to control isotype treated-mice.

**Suppl. Figure 4. Treatment with 9D1 antibody promote anti-inflammatory response in inflammatory bowel diseases model. (A)** Protein levels after *ex vivo* anti-CD3/anti-CD8 dynabeads activation at 1:2 ratio was measured by multiplex assay on day 3 after culture. Results are expressed as mean ± SEM (n≥5). **p*≤0.05 compared to control isotype treated-mice.

**Suppl. Figure 5. Treatment with 9D1 antibody reduce cytokine production after a mixed lymphocyte reaction between CD11b^+^ cells and naïve T cells.** Protein levels was validated by multiplex assay in the supernatants after 7 days of culture naïve T cells and CD11b^+^ cells at 1:2 ratio. Results are expressed as mean ± SEM (n=3). *p≤0.05, **p≤0.01, ****p*≤0.001, compared to control isotype. One-way ANOVA comparisons.

REFERENCES

BOLSTAD, B. M., IRIZARRY, R. A., ASTRAND, M. & SPEED, T. P. 2003. A comparison of normalization methods for high density oligonucleotide array data based on variance and bias. *Bioinformatics,* 19**,** 185-93.

JIANG, C. M., WANG, X. H., SHU, J., YANG, W. X., FU, P., ZHUANG, L. L. & ZHOU, G. P. 2015. Analysis of differentially expressed genes based on microarray data of glioma. *Int J Clin Exp Med,* 8**,** 17321-32.

LI, T., GAO, X., HAN, L., YU, J. & LI, H. 2018. Identification of hub genes with prognostic values in gastric cancer by bioinformatics analysis. *World J Surg Oncol,* 16**,** 114.

LIN, G. H., LIM, G., CHAN, H. L., GIANNOBILE, W. V. & WANG, H. L. 2016. Recombinant human bone morphogenetic protein 2 outcomes for maxillary sinus floor augmentation: a systematic review and meta-analysis. *Clin Oral Implants Res,* 27**,** 1349-1359.

LIN, T. H., SATO, T., BARCAY, K. R., WATERS, H., LOI, F., ZHANG, R., PAJARINEN, J., EGASHIRA, K., YAO, Z. & GOODMAN, S. B. 2015. NF-κB decoy oligodeoxynucleotide enhanced osteogenesis in mesenchymal stem cells exposed to polyethylene particle. *Tissue Eng Part A,* 21**,** 875-83.

ZAHURAK, M., PARMIGIANI, G., YU, W., SCHARPF, R. B., BERMAN, D., SCHAEFFER, E., SHABBEER, S. & COPE, L. 2007. Pre-processing Agilent microarray data. *BMC Bioinformatics,* 8**,** 142.
